# Molecular Mechanisms of *H. pylori*-Induced DNA Double-Strand Breaks

**DOI:** 10.3390/ijms19102891

**Published:** 2018-09-23

**Authors:** Dawit Kidane

**Affiliations:** Division of Pharmacology and Toxicology, College of Pharmacy, The University of Texas at Austin, Dell Pediatric Research Institute, 1400 Barbara Jordan Blvd. R1800, Austin, TX 78723, USA; dawit.kidane@austin.utexas.edu; Tel.: +1-512-495-4720; Fax: +1-512-495-4945

**Keywords:** *H. pylori*, RONS, BER, DSBs, NF-κB, NER

## Abstract

Infections contribute to carcinogenesis through inflammation-related mechanisms. *H. pylori* infection is a significant risk factor for gastric carcinogenesis. However, the molecular mechanism by which *H. pylori* infection contributes to carcinogenesis has not been fully elucidated. *H. pylori*-associated chronic inflammation is linked to genomic instability via reactive oxygen and nitrogen species (RONS). In this article, we summarize the current knowledge of *H. pylori*-induced double strand breaks (DSBs). Furthermore, we provide mechanistic insight into how processing of oxidative DNA damage via base excision repair (BER) leads to DSBs. We review recent studies on how *H. pylori* infection triggers NF-κB/inducible NO synthase (iNOS) versus NF-κB/nucleotide excision repair (NER) axis-mediated DSBs to drive genomic instability. This review discusses current research findings that are related to mechanisms of DSBs and repair during *H. pylori* infection.

## 1. Introduction

Infection contributes to 20% of cancer worldwide [1]. *H. pylori* infection is one of the most common risk factors for gastric carcinogenesis [2]. More than 50% of the human population is infected with *H. pylori*, but few develop gastric cancer [3]. Several studies have shown that *H. pylori* infection causes chronic inflammation with different degrees of pathological severity, including chronic gastritis, peptic ulcers that eventually cause gastric adenocarcinoma, and gastric mucosa-associated lymphoid tissue (MALT) lymphoma [4,5,6]. Chronic gastritis that is associated with *H. pylori* infection is the first and early stage of inflammation. When accompanied by gastric epithelial cell injury, it may contribute to gastric cancer development [7,8,9]. *H. pylori* virulence factors that contribute to host-pathogen interaction have been characterized, which increase the risk of gastric cancer pathogenesis [4,10]. These virulence factors enhance the severity of the mucosal inflammatory response, which may largely be responsible for the virulence factor-associated increased risk of gastric cancer [10].

*H. pylori* causes chronic gastritis and contributes to genotoxic activity [11,12]. However, the molecular mechanisms by which *H. pylori* promotes genotoxic activity and the host response to genotoxic factors to drive gastric carcinogenesis require more study. Based on the current knowledge, *H. pylori* infection induces a genotoxic effect via two potential mechanisms. First, *H. pylori* infection enhances the infiltration of immune cells, including neutrophils and macrophages, to produce reactive oxygen species and nitrogen species (RONS) [13]. RONS can cause DNA base damage that leads to single strand breaks (SSBs) and the enhanced expression of oncogenes [14,15,16]. Alternatively, RONS activate the oxidant-sensitive transcription factor NF-κB, which induces the expression of oncogenes and cell-cycle regulators [17,18]. Activated NF-κB is translocated to the nucleus and it forms a protein complex with NER proteins (XPG and XPF) to cleave the promoter regions of the genes and cause double strand breaks (DSBs) that impact gene expression [11].

## 2. *H. pylori* Induces Inflammation-Dependent DNA Damage

**Figure 1:**
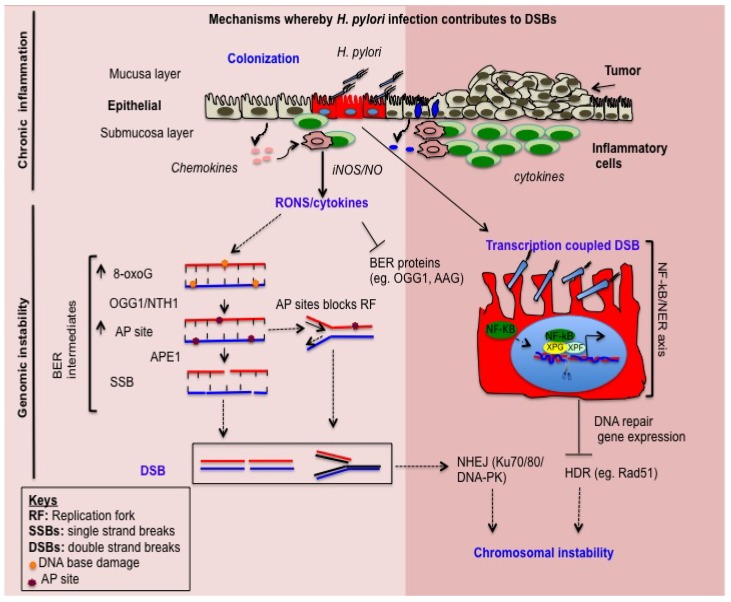
Molecular mechanisms of *H. pylori*-induced double strand breaks (DSBs). Schematic representation of how *H. pylori* induces DSBs. *H. pylori* infection causes DNA damage in gastric epithelial cells [24]. *H. pylori*-host cell interaction is a prerequisite for DSBs [25] (top panel). Persistence of the host-bacterium interaction leads to chronic inflammation and the release of inflammatory cytokines and chemokines, which contribute to oxidative DNA damage that is processed via base excision repair (BER) pathways (bottom panel). Processing oxidative DNA damage by DNA glycosylase (e.g., OGG1, NEIL1, etc.) contributes to accumulation of apurinic/apyrimidinic (AP) sites that are eventually converted to DSBs [26]. In addition, some cytokines (e.g., TNF-α) inhibit BER proteins to exacerbate genomic instability. The second pathway associated with *H. pylori*-mediated NF-κB activation leads to formation of a protein complex with nucleotide excision repair proteins (XPF and XPG), cleaves the promoter regions, and alters gene expression [11] including HR DNA repair proteins (Rad51). Alternatively, NF-κB/iNOS-mediated NO production leads to DNA damage and/or inhibits DNA repair proteins (AAG) that likely impact BER and cause DSBs. Note that solid arrow and dot arrow shows activation and alternative avenue for the down stream events respectively; T bar shows inhibition or suppression of protein activity or gene expression.

Chronic inflammation is estimated to contribute to approximately 25% of human cancers [19]. Gastric inflammation in *H. pylori* infection may be induced via two different mechanisms. The first mechanism is initiated via physical contact between the pathogen and the host epithelial cells, producing direct cell damage or enhancing the ability of epithelial cells to release pro-inflammatory mediators (Figure 1). The second mechanism is likely promoted by *H. pylori* virulence factors (e.g., *CagA*, *VacA*) that may target the potential cell signaling pathways to stimulate immune responses. Interestingly, the *H. pylori CagA* positive strain enhances chemokine activation, such as IL-8, a potent neutrophil-activating chemotactic cytokine or chemokine [20,21]. Furthermore, chemokines that are released from infected gastric epithelial cells can stimulate neutrophil infiltration and T lymphocytes to enhance RONS-mediated gastritis [22,23]. Overall, *H. pylori*-mediated gastric inflammation is associated with humoral and cell-mediated immune cells.

## 3. *H. pylori* Induces Base Excision Repair (BER) Intermediate-Dependent Double Strand Breaks (DSBs)

Chronic inflammatory conditions induce immune and epithelial cells to release RONS, which are capable of causing DNA damage and persistent cellular proliferation [27]. In addition, RONS accumulation may result in proto-oncogene activation, chromosomal aberrations, and DNA mutations [28,29]. There is considerable evidence that *H. pylori* itself induces genomic instability and epigenetic alteration in the host genome. However, there is little experimental evidence to provide mechanistic insight into how oxidative DNA damage leads to DSBs and how oxidative-damaged DNA processed via BER (Figure 1), which is thought to be the primary repair pathway against oxidative DNA damage [30]. The mechanism of *H. pylori*-induced host genomic instability remains poorly understood.

BER is crucial for maintaining genomic stability to prevent carcinogenesis [31,32,33,34]. BER is a major DNA repair pathway that removes the majority of oxidative and alkylating DNA damage without affecting the double helix DNA structure [30,35,36]. BER is the primary repair pathway of RONS-induced DNA damage during inflammation that occurs during *H. pylori* infection [37] (Table 1). Tight coordination of the different steps in BER is necessary to avoid genomic instability [38]. BER is initiated by the recognition and excision of the damaged base by specific DNA glycosylases. For example, the best characterized 8oxoG DNA lesions paired with cytosine are recognized and excised by bifunctional OGG1 glycosylase [39,40,41,42]. Subsequently, OGG1 remains bound to its abasic site (AP) and its turnover is stimulated by apurinic/apyrimidinic endonuclease1 (APE1) [43,44]. After AP site processing and end-remodeling, the single-nucleotide gap is filled by Pol β, and the nick is sealed by DNA ligase I to complete repair [45,46]. *H. pylori* can alter DNA repair gene expression and/or interfere with DNA repair activity [26,47,48]. Ding et al. reported live *H. pylori* upregulated APE1 expression in cultured gastric adenocarcinoma cell lines (AGS) and gastric epithelial cells that were isolated from uninfected human subjects [49]. Overexpressed APE1 likely interacts with other redox proteins to suppress ROS production [50]. In addition, Taller et al. show that coculture of *H. pylori* with gastric cancer cell lines induces DSBs in a contact-dependent manner [12]. DSBs in those cell lines lead to the activation of the ATM-dependent DNA damage response. *H. pylori*-induced DSBs likely cause chromosomal aberrations, such as deletions, insertions, and translocations, which are a major cause of the loss of heterozygosity.

Repair of oxidative DNA damage is critical for suppression of inflammation-associated carcinogenesis. However, host BER insufficiency caused by genetic polymorphism or loss of repair capacity likely exacerbates RONS-mediated DNA damage and cancer development [51,52,53] (Table 1). In addition, altered function of BER proteins causes aberrant function, including the processing of *H. pylori*-induced oxidative DNA damage that leads to SSBs [54] and mutation [47,48]. In vivo studies have shown that *H. pylori* infection in an OGG1 knockout mouse model enhances accumulation of 8oxoG DNA lesions and promotes resistance to inflammation [55,56,57]. In addition, loss of DNA glycosylase, such as *MYH* and alkyladenine DNA glycosylase (AAG), causes the accumulation of oxidative DNA damage lesions and promotes inflammation-associated tumor development [37,58,59]. *H. pylori* infection activates other BER proteins, such as PARP1 and enhances the inflammatory response, suggesting that the bacterium modulates the host PARP1 status to drive inflammation-associated gastric cancer [60]. However, cell culture experiments have shown that OGG1 downregulation in gastric epithelial cells decreases the formation of AP sites and suppress DSBs formation [26]. However, silencing of APE1 as part of the BER machinery failed to cause a significant level of *H. pylori*-induced DSBs [11].

DSBs are the principle cytotoxic lesions generated by *H. pylori* infection. DSBs can be caused by the accumulation of unrepaired BER intermediates in DNA replication independently and/or arise when DNA replication forks encounter BER intermediates including DNA SSBs [70,71]. Few studies have shown that accumulation of AP sites in *H. pylori*-infected human gastric epithelial cells leads to DSBs [26]. Toller et al. [12] reported that a direct bacterium-host interaction is a prerequisite to DSBs, rather than the release of DNA-damaging components. Overall, these results suggest that DSB formation is mediated by BER intermediates that are generated from a direct response of the host-bacterium interaction (Figure 1).

## 4. NF-κB-iNOS Axis-Dependent DSB Formation

*H. pylori* infection induces DNA damage in gastric epithelial cells [24]. Contact-dependent interactions between *H. pylori* bacteria and gastric epithelial cells activate intracellular signaling events that have further downstream effects via activation of the transcription factor NF-κB [72]. NF-κB activation is effected through a series of phosphorylation and transactivation events, triggering a downstream signaling pathway that contributes to gastric inflammation in *H. pylori*-infected individuals [73,74]. *H. pylori*-mediated NF-κB activation leads to the upregulated expression of a variety of inflammatory mediators, including IL-8 [75], and regulates genes that govern the innate and adaptive immune response [76,77]. However, aberrant NF-κB activation has been reported to function as a tumor promoter in inflammation-associated cancer [78,79].

Moreover, the host response to *H. pylori* infection enhances NF-κB activation in immune and epithelial cells, resulting in inducible nitric oxide synthase (iNOS) [80,81,82,83,84]. iNOS is an inflammatory mediator that causes the production of nitric oxide (NO) by immune cells, such as macrophages, linking chronic inflammation and tumorigenesis [85,86,87]. iNOS is expressed in response to bacterial endotoxins and cytokines and leads to NO production that enhances carcinogenesis [88]. iNOS-mediated NO induces oxidized DNA and leads to mutations associated with the infection [89,90] and DSBs [91]. Furthermore, NO has a biphasic effect on NF-κB to exert both pro- and anti-inflammatory actions. Although the ability of NO to directly damage DNA has been studied to a limited degree [92], its role in promoting potentially mutagenic changes in DNA has received far less attention. NO prevents NF-κB transactivation via the stabilization of IκBα [93] and nitrosate, a specific cysteine residue on the p50 subunit of NF-κB that reduces its DNA-binding capacity [94,95]. Few studies have shown that NF-κB plays a significant role in inhibition of pathogen-induced apoptosis in immune cells [96], suggesting that NF-κB may play a pro-inflammatory role to induce persistent macrophage activation. Other studies have shown that DNA repair proteins that are involved in BER and SSBs (PARP1) interact with NF-κB to facilitate the interactions with DNA to promote the expression of pro-inflammatory cytokines and enhance the activity of iNOS [97,98,99].

iNOS-mediated NO enhances inactivation of DNA repair enzymes that eventually contribute to genomic instability, leading to cancer development [86]. NO can nitrosylate thiol and tyrosine residues of the DNA repair proteins, causing loss of their function [100,101]. Thus, determining the effect of iNOS-generated NO on DNA repair proteins is scientifically important to uncover the impact of *H. pylori*-triggered iNOS-mediated DNA repair defects (Figure 1). Few studies have shown that DNA repair proteins are vulnerable to oxidative damage from NO because of their active sites, such as sulphydryl, tyrosine, and phenol side chains [102]. DNA repair enzymes, such as MGMT, FpyG, and PARP may be inactivated by NO-mediated nitrosylation of the cysteine-rich residues of the active site [103,104]. The integrity of the genome may be challenged during exposure to high concentrations of NO by direct oxidative damage to DNA and by inhibiting the DNA repair capacity of the enzyme (Table 1).

## 5. NF-κB-Nucleotide Excision Repair (NER) Axis-Dependent DSB Formation

*H. pylori* infection increases NF-κB activation to promote the inflammatory immune response [105]. NF-κB modulates many DNA repair genes to facilitate repair and generate DNA DSBs [106,107]. Endonucleases XPF and XPG are critical components of NER that are responsible for excising the damaged DNA strand to remove the DNA lesion. The endonuclease XPG cuts the DNA strand approximately 5–6 nucleotides downstream of 3′ of the DNA damage site. In addition, the ERCC1-XPF protein complex performs an incision of the DNA strand 20–22 nucleotides upstream of the 5′ end of the DNA [108,109]. Although these two endonucleases are recruited and form complexes with NF-κB to make preincision complexes, proper assembly of all the factors seems to be required for dual incision at the promoter region of a given gene. XPF/XPG-mediated DSBs amplify NF-κB target inflammatory gene expression and promote host cell survival [11]. The NF-κB complex in XPG and XPF in the formation of the active DSBs at the chromatin region of the genome likely promotes a hub that controls gene expression (Figure 1). Furthermore, the expression level of XPG is significantly associated with an *H. pylori*-positive sample [110] (Table 1). However, silencing XPG strongly reduced the DSBs upon *H. pylori* infection, suggesting that NER-dependent DSBs contribute to genomic instability during infection. *H. pylori* infection modulates NER and enhances the interaction with NF-κB, likely providing a molecular basis for insights into how *H. pylori* infection induces transcription-associated DSBs (Figure 1).

## 6. *H. pylori* Impairs DSBs Repair

DSBs can be repaired via two major repair pathways [111,112]. Homologous recombination (HR) requires sequence homology of extensive DNA regions from an undamaged sister chromatid or homologous chromosomes. In contrast, nonhomologous DNA end-joining (NHEJ) occurs throughout the cell cycles and is processed without any sequence homology or few end homology sequences. *H. pylori*-induced DSBs are likely recognized by the MRE11-RAD50-NBS1 (MRN) complex [113,114], which is recognized and processed the DNA ends, resulting in the activation of ataxia telangiectasia mutated kinase (ATM) [115,116]. ATM is a major DNA damage response sensor of DSBs. It directly binds to the damaged DNA and phosphorylates target proteins, including H2AX protein at serine 139 of the histone (γH2AX) to mark DSBs sites [116,117,118,119]. Alternatively, ATM is involved in mediating the NF-κB response to DSBs [120].

In the NHEJ pathway, DNA-dependent protein kinase (DNA-PK) and Ku proteins play key roles in mediation of incompatible DNA ends. DNA-PK may function as a DNA damage sensor or scaffolding to assemble repair proteins including Ku proteins to bind the two ends of the break together. Then, ligase IV/XRCC4/XLF carries out the ligation reaction [121] to complete NHEJ repair. However, *H. pylori* causes an increase in Ku70/80, which may indicate that NHEJ-mediated repair contributes to genomic instability [69]. Recent evidence has shown that altered DNA-PK and Ku70/80 are associated with pathological processes in different types of cancer [122]. Moreover, Ku70 and DNA-PK are expressed in *H. pylori*-associated gastritis, intestinal metaplasia and gastric adenoma tissues [68]. Furthermore, Lim et al. [123] showed that activated NF-κB-Cox2 axis plays a significant role in enhancing the expression of KU70/80. In contrast, the loss of Ku proteins leads to an accumulation of DNA damage that eventually causes cell death in gastric epithelial cells [124].

Our previous study shows that DSBs significantly increase in the G1 stage of the cell cycle after *H. pylori* infection [26], suggesting that NHEJ repair might be involved in promotion of error-prone repair. When DSBs are generated during the S phase at DNA replication forks or after replication in the G2 phase of the cell cycle, HR may contribute to genome integrity (Figure 1). Few studies have shown that NF-κB interacts with HR proteins (e.g., CtIP-BRCA1 complexes) to stabilize BRCA1 and stimulate HR-mediated repair [125] Activation of multiple molecular targets by NF-κB enhances rapid activation of HR and may permit the accelerated proliferation of cells. However, *H. pylori* infection-mediated activation of the NF-κB/NER axis causes defects in HR that reduce the ability of DSB repair [11]. *H. pylori* infection modulates DSB repair efficiency to exacerbate genomic instability and facilitate gastric carcinogenesis.

## 7. Summary

*H. pylori* infection is a contributing factor for gastric cancer. This review highlights how *H. pylori*-associated DNA base damage in infected host cells is likely processed via BER to generate DSBs. In addition, this review provides a comprehensive overview of how *H. pylori*-associated DSBs are induced via the NF-κB/NER axis and NF-κB/iNOS axis, influencing DNA repair gene expression and enhancing genomic instability and carcinogenesis (Figure 1). Several studies have shown that *H. pylori* induces DSB and promotes gastric carcinogenesis. Host BER, NER, and NHEJ genetic variants could modify the process of carcinogenesis in *H. pylori* infected hosts. Alteration of the activity of enzymes that function in DNA repair as a result of genetic mutation could significantly impact gastric cancer risk. Other studies are needed to uncover the associations of the BER, NER and NHEJ genetic variants with increased susceptibility to gastric cancer in *H. pylori*-infected hosts. In addition, many questions remain regarding the mechanism of DSB formation and how breaks are processed via the NHEJ or HR pathways. Does *H. pylori* infection decrease tumor latency for carriers of BER variant genotypes? Future studies will likely explore how *H. pylori* manipulates host DNA repair genetics and how NHEJ processes DSBs. Therefore, the relevant DNA repair of the host genetics and *H. pylori* infection status of the host should be considered in studies of gastric cancer susceptibility in the future.

## Figures and Tables

**Table 1:** Interplay between *H. pylori* and relevant DNA repair gene products.

Gene	Role of Gene Products	Interplay between *H. pylori* & Gene	References
*BER*			
OGG1	removes 8oxoG and FapyG DNA lesions	absence causes increased mutation frequency, fewer DSBs and decreased inflammation	[26,55,61]
NEIL1	removes 8oxoG and Tg lesions	decreases mRNA in tumor; unknown role during infection	[34]
APE1	acts as a negative regulator of ROS and enhances chemokine release	enhances the expression of mRNA and protein	[49,62,63]
POLB	removes 5′-dRP group and adds a single nucleotide base	infection does not affect gene expression and protein level	[26]
XRCC1	scaffold protein enhance ligation	downregulated via promoter hypermethylation	[64,65]
*NER*			
XPG	cuts the 3′ of the DNA damage site; forms complex with NF-κB and promotes target gene expression	moderates change in gene expression	[11,66,67]
XPF	forms complex with NF-κB & promotes targeted gene expression	moderates change in gene expression	[11,66]
XPA	recognition bulk DNA adduct	increases IL-8 cytokine expression	[11,66]
*NHEJ*			
DNA-PK	increases cellular proliferation & facilitates NHEJ (nonhomologous DNA end-joining) repair	enhances activity and expression	[68]
Ku70/80	protects DNA DSB ends and prevents cell death	decreases protein level	[69]
DNA ligase IV	completes NHEJ repair by sealing DNA DSB regions	knock-down enhances DSBs	[11]
XRCC4	scaffold to hold DNA DSBs ends to enhance ligation	knock-down promotes DNA DSBs	[11]
*HR*			
*NBS1*	DNA DSB end processing/DDR	decreases expression and may impair DNA end processing and DDR	[66]
*Rad51*	strand exchange and enhances DSB repair	decreases gene expression; however, infection does not increase DSBs	[25]
*RPA1*	ssDNA binding and DDR	downregulates mRNA	[66]
*Mre11*	DSB end processing and DDR	decreases expression and impairs end processing and DDR	[66]

## References

[1] Kuper H., Adami H.O., Trichopoulos D. (2000). Infections as a major preventable cause of human cancer. J. Int. Med..

[2] De Martel C., Forman D., Plummer M. (2013). Gastric cancer: Epidemiology and risk factors. Gastroenterol. Clin..

[3] Peek R.M., Blaser M.J. (2002). *Helicobacter pylori* and gastrointestinal tract adenocarcinomas. Nat. Rev. Cancer.

[4] Covacci A., Telford J.L., Del Giudice G., Parsonnet J., Rappuoli R. (1999). Helicobacter pylori virulence and genetic geography. Science.

[5] Montecucco C., Rappuoli R. (2001). Living dangerously: How *Helicobacter pylori* survives in the human stomach. Nat. Rev. Mol. Cell Biol..

[6] Monack D.M., Mueller A., Falkow S. (2004). Persistent bacterial infections: The interface of the pathogen and the host immune system. Nat. Rev. Microbiol..

[7] Correa P. (1992). Human gastric carcinogenesis: A multistep and multifactorial process—First american cancer society award lecture on cancer epidemiology and prevention. Cancer Res..

[8] Ohnishi N., Yuasa H., Tanaka S., Sawa H., Miura M., Matsui A., Higashi H., Musashi M., Iwabuchi K., Suzuki M. (2008). Transgenic expression of *Helicobacter pylori* CagA induces gastrointestinal and hematopoietic neoplasms in mouse. Proc. Natl. Acad. Sci. USA.

[9] Smoot D.T., Wynn Z., Elliott T.B., Allen C.R., Mekasha G., Naab T., Ashktorab H. (1999). Effects of *Helicobacter pylori* on proliferation of gastric epithelial cells in vitro. Am. J. Gastroenterol..

[10] Dubreuil J.D., Giudice G.D., Rappuoli R. (2002). *Helicobacter pylori* interactions with host serum and extracellular matrix proteins: Potential role in the infectious process. Microbiol. Mol. Biol. Rev..

[11] Hartung M.L., Gruber D.C., Koch K.N., Gruter L., Rehrauer H., Tegtmeyer N., Backert S., Muller A. (2015). *H. Pylori*-induced DNA strand breaks are introduced by nucleotide excision repair endonucleases and promote NF-κB target gene expression. Cell Rep..

[12] Toller I.M., Neelsen K.J., Steger M., Hartung M.L., Hottiger M.O., Stucki M., Kalali B., Gerhard M., Sartori A.A., Lopes M. (2011). Carcinogenic bacterial pathogen *Helicobacter pylori* triggers DNA double-strand breaks and a DNA damage response in its host cells. Proc. Natl. Acad. Sci. USA.

[13] Suzuki M., Miura S., Mori M., Kai A., Suzuki H., Fukumura D., Suematsu M., Tsuchiya M. (1994). Rebamipide, a novel antiulcer agent, attenuates *Helicobacter pylori* induced gastric mucosal cell injury associated with neutrophil derived oxidants. Gut.

[14] Cerutti P.A. (1994). Oxy-radicals and cancer. Lancet.

[15] Feig D.I., Reid T.M., Loeb L.A. (1994). Reactive oxygen species in tumorigenesis. Cancer Res..

[16] Schreck R.R. (1992). Tumor suppressor gene (*Rb* and *p53*) mutations in osteosarcoma. Ped. Hematol. Oncol..

[17] D’Angio C.T., Finkelstein J.N. (2000). Oxygen regulation of gene expression: A study in opposites. Mol. Genet. MeTable.

[18] Adler V., Yin Z., Tew K.D., Ronai Z. (1999). Role of redox potential and reactive oxygen species in stress signaling. Oncogene.

[19] Kawanishi S., Ohnishi S., Ma N., Hiraku Y., Murata M. (2017). Crosstalk between DNA damage and inflammation in the multiple steps of carcinogenesis. Int. J. Mol. Sci..

[20] Eck M., Schmausser B., Scheller K., Toksoy A., Kraus M., Menzel T., Muller-Hermelink H.K., Gillitzer R. (2000). CXC chemokines Groα/IL-8 and IP-10/MIG in *Helicobacter pylori* gastritis. Clin. Exp. Immunol..

[21] Watanabe N., Shimada T., Ohtsuka Y., Hiraishi H., Terano A. (1997). Proinflammatory cytokines and *Helicobacter pylori* stimulate CC-chemokine expression in gastric epithelial cells. J. Physiol. Pharmacol..

[22] Nozawa Y., Nishihara K., Peek R.M., Nakano M., Uji T., Ajioka H., Matsuura N., Miyake H. (2002). Identification of a signaling cascade for interleukin-8 production by *Helicobacter pylori* in human gastric epithelial cells. Biochem. Pharmacol..

[23] Naito Y., Yoshikawa T. (2002). Molecular and cellular mechanisms involved in *Helicobacter pylori*-induced inflammation and oxidative stress. Free Radic. Biol. Med..

[24] Obst B., Wagner S., Sewing K.F., Beil W. (2000). *Helicobacter pylori* causes DNA damage in gastric epithelial cells. Carcinogenesis.

[25] Hanada K., Uchida T., Tsukamoto Y., Watada M., Yamaguchi N., Yamamoto K., Shiota S., Moriyama M., Graham D.Y., Yamaoka Y. (2014). *Helicobacter pylori* infection introduces DNA double-strand breaks in host cells. Infect. Immun..

[26] Kidane D., Murphy D.L., Sweasy J.B. (2014). Accumulation of abasic sites induces genomic instability in normal human gastric epithelial cells during *Helicobacter pylori* infection. Oncogenesis.

[27] Perryman S.V., Sylvester K.G. (2006). Repair and regeneration: Opportunities for carcinogenesis from tissue stem cells. J. Cell Mol. Med..

[28] Floyd R.A. (1990). Role of oxygen free radicals in carcinogenesis and brain ischemia. FASEB J..

[29] Du M.Q., Carmichael P.L., Phillips D.H. (1994). Induction of activating mutations in the human c-Ha-*ras*-1 proto-oncogene by oxygen free radicals. Mol Carcinog.

[30] Dianov G.L., Hubscher U. (2013). Mammalian base excision repair: The forgotten archangel. Nucleic Acids Res..

[31] Al-Tassan N., Chmiel N.H., Maynard J., Fleming N., Livingston A.L., Williams G.T., Hodges A.K., Davies D.R., David S.S., Sampson J.R. (2002). Inherited variants of *MYH* associated with somatic g:C → t:A mutations in colorectal tumors. Nat. Genet..

[32] Farrington S.M., Tenesa A., Barnetson R., Wiltshire A., Prendergast J., Porteous M., Campbell H., Dunlop M.G. (2005). Germline susceptibility to colorectal cancer due to base-excision repair gene defects. Am. J. Hum. Genet..

[33] Mahjabeen I., Masood N., Baig R.M., Sabir M., Inayat U., Malik F.A., Kayani M.A. (2012). Novel mutations of OGG1 base excision repair pathway gene in laryngeal cancer patients. Fam. Cancer.

[34] Shinmura K., Tao H., Goto M., Igarashi H., Taniguchi T., Maekawa M., Takezaki T., Sugimura H. (2004). Inactivating mutations of the human base excision repair gene *NEIL1* in gastric cancer. Carcinogenesis.

[35] Kim Y.J., Wilson D.M. (2012). Overview of base excision repair biochemistry. Curr. Mol. Pharmacol..

[36] Wallace S.S., Murphy D.L., Sweasy J.B. (2012). Base excision repair and cancer. Cancer Lett..

[37] Meira L.B., Bugni J.M., Green S.L., Lee C.W., Pang B., Borenshtein D., Rickman B.H., Rogers A.B., Moroski-Erkul C.A., McFaline J.L. (2008). DNA damage induced by chronic inflammation contributes to colon carcinogenesis in mice. J. Clin. Investig..

[38] Allinson S.L., Sleeth K.M., Matthewman G.E., Dianov G.L. (2004). Orchestration of base excision repair by controlling the rates of enzymatic activities. DNA Repair.

[39] Radicella J.P., Dherin C., Desmaze C., Fox M.S., Boiteux S. (1997). Cloning and characterization of *hOGG1*, a human homolog of the *OGG1* gene of *Saccharomyces cerevisiae*. Proc. Natl. Acad. Sci. USA.

[40] Aburatani H., Hippo Y., Ishida T., Takashima R., Matsuba C., Kodama T., Takao M., Yasui A., Yamamoto K., Asano M. (1997). Cloning and characterization of mammalian 8-hydroxyguanine-specific DNA glycosylase/apurinic, apyrimidinic lyase, a functional mutm homologue. Cancer Res..

[41] Fortini P., Parlanti E., Sidorkina O.M., Laval J., Dogliotti E. (1999). The type of DNA glycosylase determines the base excision repair pathway in mammalian cells. J. Biol. Chem..

[42] Nishimura S. (2002). Involvement of mammalian OGG1 (MMH) in excision of the 8-hydroxyguanine residue in DNA. Free Radic. Biol. Med..

[43] Mokkapati S.K., Wiederhold L., Hazra T.K., Mitra S. (2004). Stimulation of DNA glycosylase activity of OGG1 by NEIL1: Functional collaboration between two human DNA glycosylases. Biochemistry.

[44] Sidorenko V.S., Nevinsky G.A., Zharkov D.O. (2007). Mechanism of interaction between human 8-oxoguanine-DNA glycosylase and ap endonuclease. DNA Repair.

[45] Fortini P., Pascucci B., Parlanti E., D’Errico M., Simonelli V., Dogliotti E. (2003). The base excision repair: Mechanisms and its relevance for cancer susceptibility. Biochimie.

[46] Robertson A.B., Klungland A., Rognes T., Leiros I. (2009). DNA repair in mammalian cells: Base excision repair: The long and short of it. Cell. Mol. Life Sci..

[47] Park D.I., Park S.H., Kim S.H., Kim J.W., Cho Y.K., Kim H.J., Sohn C.I., Jeon W.K., Kim B.I., Cho E.Y. (2005). Effect of *Helicobacter pylori* infection on the expression of DNA mismatch repair protein. Helicobacter.

[48] Kim J.J., Tao H., Carloni E., Leung W.K., Graham D.Y., Sepulveda A.R. (2002). *Helicobacter pylori* impairs DNA mismatch repair in gastric epithelial cells. Gastroenterology.

[49] Ding S.Z., O’Hara A.M., Denning T.L., Dirden-Kramer B., Mifflin R.C., Reyes V.E., Ryan K.A., Elliott S.N., Izumi T., Boldogh I. (2004). Helicobacter pylori and h2o2 increase ap endonuclease-1/redox factor-1 expression in human gastric epithelial cells. Gastroenterology.

[50] Den Hartog G., Chattopadhyay R., Ablack A., Hall E.H., Butcher L.D., Bhattacharyya A., Eckmann L., Harris P.R., Das S., Ernst P.B. (2016). Regulation of Rac1 and reactive oxygen species production in response to infection of gastrointestinal epithelia. PLoS Pathog..

[51] Teoule R., Bert C., Bonicel A. (1977). Thymine fragment damage retained in the DNA polynucleotide chain after gamma irradiation in aerated solutions. II. Radiat. Res..

[52] Altieri F., Grillo C., Maceroni M., Chichiarelli S. (2008). DNA damage and repair: From molecular mechanisms to health implications. Antioxid. Redox Signal..

[53] Grollman A.P., Moriya M. (1993). Mutagenesis by 8-oxoguanine: An enemy within. Trends Genet..

[54] Cooke M.S., Evans M.D., Dizdaroglu M., Lunec J. (2003). Oxidative DNA damage: Mechanisms, mutation, and disease. FASEB J..

[55] Klungland A., Rosewell I., Hollenbach S., Larsen E., Daly G., Epe B., Seeberg E., Lindahl T., Barnes D.E. (1999). Accumulation of premutagenic DNA lesions in mice defective in removal of oxidative base damage. Proc. Natl. Acad. Sci. USA.

[56] Minowa O., Arai T., Hirano M., Monden Y., Nakai S., Fukuda M., Itoh M., Takano H., Hippou Y., Aburatani H. (2000). *Mmh*/*Ogg1* gene inactivation results in accumulation of 8-hydroxyguanine in mice. Proc. Natl. Acad. Sci. USA.

[57] Touati E., Michel V., Thiberge J.M., Ave P., Huerre M., Bourgade F., Klungland A., Labigne A. (2006). Deficiency in OGG1 protects against inflammation and mutagenic effects associated with *H. pylori* infection in mouse. Helicobacter.

[58] Cheadle J.P., Dolwani S., Sampson J.R. (2003). Inherited defects in the DNA glycosylase MYH cause multiple colorectal adenoma and carcinoma. Carcinogenesis.

[59] Sakamoto K., Tominaga Y., Yamauchi K., Nakatsu Y., Sakumi K., Yoshiyama K., Egashira A., Kura S., Yao T., Tsuneyoshi M. (2007). Mutyh-null mice are susceptible to spontaneous and oxidative stress induced intestinal tumorigenesis. Cancer Res..

[60] Nossa C.W., Blanke S.R. (2010). Helicobacter pylori activation of PARP-1: Usurping a versatile regulator of host cellular health. Gut Microbes.

[61] Mabley J.G., Pacher P., Deb A., Wallace R., Elder R.H., Szabo C. (2005). Potential role for 8-oxoguanine DNA glycosylase in regulating inflammation. FASEB J..

[62] Bhattacharyya A., Chattopadhyay R., Burnette B.R., Cross J.V., Mitra S., Ernst P.B., Bhakat K.K., Crowe S.E. (2009). Acetylation of apurinic/apyrimidinic endonuclease-1 regulates *Helicobacter pylori*-mediated gastric epithelial cell apoptosis. Gastroenterology.

[63] O’Hara A.M., Bhattacharyya A., Mifflin R.C., Smith M.F., Ryan K.A., Scott K.G., Naganuma M., Casola A., Izumi T., Mitra S. (2006). Interleukin-8 induction by helicobacter pylori in gastric epithelial cells is dependent on apurinic/apyrimidinic endonuclease-1/redox factor-1. J. Immunol..

[64] Wang P., Tang J.T., Peng Y.S., Chen X.Y., Zhang Y.J., Fang J.Y. (2010). Xrcc1 downregulated through promoter hypermethylation is involved in human gastric carcinogenesis. J. Dig. Dis..

[65] Cannan W.J., Rashid I., Tomkinson A.E., Wallace S.S., Pederson D.S. (2017). The human ligase iiialpha-xrcc1 protein complex performs DNA nick repair after transient unwrapping of nucleosomal DNA. J. Biol. Chem..

[66] Koeppel M., Garcia-Alcalde F., Glowinski F., Schlaermann P., Meyer T.F. (2015). *Helicobacter pylori* infection causes characteristic DNA damage patterns in human cells. Cell Rep..

[67] Klungland A., Hoss M., Gunz D., Constantinou A., Clarkson S.G., Doetsch P.W., Bolton P.H., Wood R.D., Lindahl T. (1999). Base excision repair of oxidative DNA damage activated by XPG protein. Mol. Cell.

[68] Lee H.S., Choe G., Park K.U., Park D.J., Yang H.K., Lee B.L., Kim W.H. (2007). Altered expression of DNA-dependent protein kinase catalytic subunit (DNA-PKcs) during gastric carcinogenesis and its clinical implications on gastric cancer. Int. J. Oncol..

[69] Bae M., Lim J.W., Kim H. (2013). Oxidative DNA damage response in *Helicobacter pylori*-infected mongolian gerbils. J. Cancer Prev..

[70] Khanna K.K., Jackson S.P. (2001). DNA double-strand breaks: Signaling, repair and the cancer connection. Nat. Genet..

[71] Mills K.D., Ferguson D.O., Alt F.W. (2003). The role of DNA breaks in genomic instability and tumorigenesis. Immunol. Rev..

[72] Maeda S., Yoshida H., Ogura K., Mitsuno Y., Hirata Y., Yamaji Y., Akanuma M., Shiratori Y., Omata M. (2000). *H. Pylori* activates NF-κB through a signaling pathway involving IκB kinases, NF-κB -inducing kinase, TRAF2, and TRAF6 in gastric cancer cells. Gastroenterology.

[73] Bhattacharyya A., Pathak S., Kundu M., Basu J. (2002). Mitogen-activated protein kinases regulate mycobacterium avium-induced tumor necrosis factor-α release from macrophages. FEMS Immunol. Med. Microbiol..

[74] Hayden M.S., Ghosh S. (2008). Shared principles in NF-κB signaling. Cell.

[75] De Luca A., Iaquinto G. (2004). *Helicobacter pylori* and gastric diseases: A dangerous association. Cancer Lett..

[76] Lamb A., Chen L.F. (2010). The many roads traveled by *Helicobacter pylori* to NF-κB activation. Gut Microbes.

[77] Orlowski R.Z., Baldwin A.S. (2002). NF-κB as a therapeutic target in cancer. Trends Mol. Med..

[78] Pikarsky E., Porat R.M., Stein I., Abramovitch R., Amit S., Kasem S., Gutkovich-Pyest E., Urieli-Shoval S., Galun E., Ben-Neriah Y. (2004). NF-κB functions as a tumour promoter in inflammation-associated cancer. Nature.

[79] Oussaief L., Ramirez V., Hippocrate A., Arbach H., Cochet C., Proust A., Raphael M., Khelifa R., Joab I. (2011). NF-κB-mediated modulation of inducible nitric oxide synthase activity controls induction of the epstein-barr virus productive cycle by transforming growth factor β1. J. Virol..

[80] Viala J., Chaput C., Boneca I.G., Cardona A., Girardin S.E., Moran A.P., Athman R., Memet S., Huerre M.R., Coyle A.J. (2004). Nod1 responds to peptidoglycan delivered by the *Helicobacter pylori* cag pathogenicity island. Nat. Immunol..

[81] Brandt S., Kwok T., Hartig R., Konig W., Backert S. (2005). NF-κB activation and potentiation of proinflammatory responses by the *Helicobacter pylori* caga protein. Proc. Natl. Acad. Sci. USA.

[82] Geem D., Medina-Contreras O., Kim W., Huang C.S., Denning T.L. (2012). Isolation and characterization of dendritic cells and macrophages from the mouse intestine. J. Vis. Exp..

[83] Geller D.A., Di Silvio M., Nussler A.K., Wang S.C., Shapiro R.A., Simmons R.L., Billiar T.R. (1993). Nitric oxide synthase expression is induced in hepatocytes in vivo during hepatic inflammation. J. Surg. Res..

[84] Nussler A.K., Geller D.A., Sweetland M.A., Di Silvio M., Billiar T.R., Madariaga J.B., Simmons R.L., Lancaster J.R. (1993). Induction of nitric oxide synthesis and its reactions in cultured human and rat hepatocytes stimulated with cytokines plus LPS. Biochem. Biophys. Res. Commun..

[85] Lowenstein C.J., Padalko E. (2004). iNOS (NOS2) at a glance. J. Cell Sci..

[86] Choudhari S.K., Chaudhary M., Bagde S., Gadbail A.R., Joshi V. (2013). Nitric oxide and cancer: A review. World J. Surg. Oncol..

[87] Thomsen L.L., Lawton F.G., Knowles R.G., Beesley J.E., Riveros-Moreno V., Moncada S. (1994). Nitric oxide synthase activity in human gynecological cancer. Cancer Res..

[88] Rieder G., Hofmann J.A., Hatz R.A., Stolte M., Enders G.A. (2003). Up-regulation of inducible nitric oxide synthase in *Helicobacter pylori*-associated gastritis may represent an increased risk factor to develop gastric carcinoma of the intestinal type. Int. J. Med. Microbiol..

[89] Touati E., Michel V., Thiberge J.M., Wuscher N., Huerre M., Labigne A. (2003). Chronic *Helicobacter pylori* infections induce gastric mutations in mice. Gastroenterology.

[90] Wink D.A., Kasprzak K.S., Maragos C.M., Elespuru R.K., Misra M., Dunams T.M., Cebula T.A., Koch W.H., Andrews A.W., Allen J.S. (1991). DNA deaminating ability and genotoxicity of nitric oxide and its progenitors. Science.

[91] Baydoun H.H., Cherian M.A., Green P., Ratner L. (2015). Inducible nitric oxide synthase mediates DNA double strand breaks in human T-cell leukemia virus type 1-induced leukemia/lymphoma. Retrovirology.

[92] Nguyen T., Brunson D., Crespi C.L., Penman B.W., Wishnok J.S., Tannenbaum S.R. (1992). DNA damage and mutation in human cells exposed to nitric oxide in vitro. Proc. Natl. Acad. Sci. USA.

[93] Peng H.B., Libby P., Liao J.K. (1995). Induction and stabilization of IκBα by nitric oxide mediates inhibition of NF-κB. J. Biol. Chem..

[94] Matthews J.R., Botting C.H., Panico M., Morris H.R., Hay R.T. (1996). Inhibition of NF-κB DNA binding by nitric oxide. Nucleic Acids Res..

[95] Schroeder R.A., Punzalan C., Kuo P.C. (1999). Endotoxin-mediated S-nitrosylation of p50 alters NF-κB -dependent gene transcription in ANA-1 murine macrophages. J. Immunol..

[96] Park J.M., Greten F.R., Wong A., Westrick R.J., Arthur J.S., Otsu K., Hoffmann A., Montminy M., Karin M. (2005). Signaling pathways and genes that inhibit pathogen-induced macrophage apoptosis—CREB and NF-κB as key regulators. Immunity.

[97] Hassa P.O., Covic M., Hasan S., Imhof R., Hottiger M.O. (2001). The enzymatic and DNA binding activity of PARP-1 are not required for NF-κB coactivator function. J. Biol. Chem..

[98] Ullrich O., Diestel A., Eyupoglu I.Y., Nitsch R. (2001). Regulation of microglial expression of integrins by poly (ADP-ribose) polymerase-1. Nat. Cell Biol..

[99] Wang J., Hao L., Wang Y., Qin W., Wang X., Zhao T., Liu Y., Sheng L., Du Y., Zhang M. (2015). Inhibition of poly (ADP-ribose) polymerase and inducible nitric oxide synthase protects against ischemic myocardial damage by reduction of apoptosis. Mol. Med. Rep..

[100] Ischiropoulos H., Zhu L., Beckman J.S. (1992). Peroxynitrite formation from macrophage-derived nitric oxide. Arch. Biochem. Biophys..

[101] Kong S.K., Yim M.B., Stadtman E.R., Chock P.B. (1996). Peroxynitrite disables the tyrosine phosphorylation regulatory mechanism: Lymphocyte-specific tyrosine kinase fails to phosphorylate nitrated cdc2(6-20)NH2 peptide. Proc. Natl. Acad. Sci. USA.

[102] Starke D.W., Chen Y., Bapna C.P., Lesnefsky E.J., Mieyal J.J. (1997). Sensitivity of protein sulfhydryl repair enzymes to oxidative stress. Free Radic. Biol. Med..

[103] Wink D.A., Laval J. (1994). The Fpg protein, a DNA repair enzyme, is inhibited by the biomediator nitric oxide in vitro and in vivo. Carcinogenesis.

[104] O’Connor T.R., Graves R.J., de Murcia G., Castaing B., Laval J. (1993). Fpg protein of escherichia coli is a zinc finger protein whose cysteine residues have a structural and/or functional role. J. Biol. Chem..

[105] Telford J.L., Covacci A., Rappuoli R., Chiara P. (1997). Immunobiology of helicobacter pylori infection. Curr. Opin. Immunol..

[106] Le May N., Fradin D., Iltis I., Bougneres P., Egly J.M. (2012). XPG and XPF endonucleases trigger chromatin looping and DNA demethylation for accurate expression of activated genes. Mol. Cell.

[107] Le May N., Mota-Fernandes D., Velez-Cruz R., Iltis I., Biard D., Egly J.M. (2010). NER factors are recruited to active promoters and facilitate chromatin modification for transcription in the absence of exogenous genotoxic attack. Mol. Cell.

[108] Leibeling D., Laspe P., Emmert S. (2006). Nucleotide excision repair and cancer. J. Mol. Histol..

[109] Friedberg E.C. (2001). How nucleotide excision repair protects against cancer. Nat. Rev. Cancer.

[110] Deng N., Liu J.W., Sun L.P., Xu Q., Duan Z.P., Dong N.N., Yuan Y. (2014). Expression of XPG protein in the development, progression and prognosis of gastric cancer. PLoS ONE.

[111] Ivanov E.L., Haber J.E. (1997). DNA repair: Rad alert. Curr. Biol..

[112] Kanaar R., Hoeijmakers J.H. (1997). Recombination and joining: Different means to the same ends. Genes Funct..

[113] Matsuura S., Tauchi H., Nakamura A., Kondo N., Sakamoto S., Endo S., Smeets D., Solder B., Belohradsky B.H., Der Kaloustian V.M. (1998). Positional cloning of the gene for Nijmegen breakage syndrome. Nat. Genet..

[114] Carney J.P., Maser R.S., Olivares H., Davis E.M., Le Beau M., Yates J.R., Hays L., Morgan W.F., Petrini J.H. (1998). The hmre11/hrad50 protein complex and nijmegen breakage syndrome: Linkage of double-strand break repair to the cellular DNA damage response. Cell.

[115] Stracker T.H., Petrini J.H. (2011). The mre11 complex: Starting from the ends. Nat. Rev. Mol. Cell Biol..

[116] Shiloh Y., Lehmann A.R. (2004). Maintaining integrity. Nat. Cell Biol..

[117] Bakkenist C.J., Kastan M.B. (2004). Phosphatases join kinases in DNA-damage response pathways. Trends Cell Biol..

[118] Dupre A., Boyer-Chatenet L., Gautier J. (2006). Two-step activation of ATM by DNA and the Mre11–Rad50–Nbs1 complex. Nat. Struct. Mol. Biol..

[119] Burma S., Chen B.P., Murphy M., Kurimasa A., Chen D.J. (2001). ATM phosphorylates histone H2AX in response to DNA double-strand breaks. J. Biol. Chem..

[120] Li N., Banin S., Ouyang H., Li G.C., Courtois G., Shiloh Y., Karin M., Rotman G. (2001). ATM is required for IκB kinase (IKKk) activation in response to DNA double strand breaks. J. Biol. Chem..

[121] Ahnesorg P., Smith P., Jackson S.P. (2006). XLF interacts with the XRCC4-DNA ligase IV complex to promote DNA nonhomologous end-joining. Cell.

[122] Abe T., Ishiai M., Hosono Y., Yoshimura A., Tada S., Adachi N., Koyama H., Takata M., Takeda S., Enomoto T. (2008). Ku70/80, DNA-PKcs, and artemis are essential for the rapid induction of apoptosis after massive DSB formation. Cell. Signal..

[123] Lim J.W., Kim H., Kim K.H. (2002). Expression of Ku70 and Ku80 mediated by NF-κB and cyclooxygenase-2 is related to proliferation of human gastric cancer cells. J. Biol. Chem..

[124] Song J.Y., Lim J.W., Kim H., Morio T., Kim K.H. (2003). Oxidative stress induces nuclear loss of DNA repair proteins Ku70 and Ku80 and apoptosis in pancreatic acinar AR42J cells. J. Biol. Chem..

[125] Volcic M., Karl S., Baumann B., Salles D., Daniel P., Fulda S., Wiesmuller L. (2012). NF-κB regulates DNA double-strand break repair in conjunction with BRCA1–CtIP complexes. Nucleic Acids Res..

